# Prevalence of pre-iridal monocellular and fibrovascular membranes in canine globes affected with congenital glaucoma associated with anterior segment dysgenesis, primary glaucoma associated with goniodysgenesis, and secondary glaucoma

**DOI:** 10.3389/fvets.2024.1289283

**Published:** 2024-02-20

**Authors:** Leila Bedos, Lynne Sandmeyer, John Campbell, Bruce H. Grahn

**Affiliations:** ^1^Department of Veterinary Clinical Sciences, Iowa State University, Ames, IA, United States; ^2^Departments of Small Animal Clinical Sciences and Large Animal Clinical Sciences, Western College of Veterinary Medicine, University of Saskatchewan, Saskatoon, SK, Canada

**Keywords:** dogs, eye, glaucoma, pre-iridal monocellular and fibrovascular membranes, goniodysgenesis, congenital/ASD-associated glaucoma, secondary glaucoma, primary glaucoma associated with goniodysgenesis

## Abstract

**Objectives:**

The objectives of this study were to (i) evaluate the prevalence of pre-iridal monocellular and fibrovascular membranes in canine globes affected with congenital glaucoma associated with anterior segment dysgenesis (ASD), primary glaucoma associated with goniodysgenesis (GD), and secondary glaucoma, and (ii) examine the associations between monocellular and fibrovascular membranes by breed, gender, age and histopathologic ocular changes on light microscopic examination.

**Methods:**

Records of dogs who had eyes enucleated due to blindness and uncontrolled glaucoma were reviewed. Glaucoma was categorized clinically and histologically into three groups: congenital/ASD, primary/GD, and secondary glaucoma. The presence or absence and type of pre-iridal membrane (monocellular or fibrovascular) and other intraocular histologic findings were reviewed and compared statistically for each group.

**Results:**

In total, 108 canine globes (101 dogs) were included. Pre-iridal monocellular membranes were identified with light microscopy in 10 out of 19 congenital/ASD, 29 out of 40 primary, and 23 out of 49 secondary glaucoma globes. Fibrovascular membranes were observed in 3 out of 19 congenital/ASD, 9 out of 40 in primary, and 24 out of 49 secondary glaucoma globes. There were no associations between the type of membrane and breed, gender, or age. Peripheral anterior synechiae were more common in globes with fibrovascular membranes, and uveal atrophy was more common in globes with monocellular membranes.

**Conclusion:**

Pre-iridal monocellular membranes are common in all types of canine glaucoma. They are identified with light microscopy most easily in cases of primary glaucoma, and they are masked by pre-iridal fibrovascular membranes in other forms of glaucoma.

## Introduction

Glaucoma is a common condition in veterinary ophthalmology characterized by elevated intraocular pressure (IOP) that induces significant ocular dysfunction and degeneration of all ocular tissues with buphthalmos and blindness as common sequelae (1, 2). Although the pathogenesis of glaucoma is not fully understood, elevated IOP develops due to inadequate aqueous humor filtration out of the globe, which leads to progressive retinal and optic nerve degeneration related to axonal damage caused by apoptosis and necrosis of retinal ganglion cells (3, 4). In dogs, aqueous humor outflow occurs primarily through the iridocorneal angle (conventional outflow), but a small percentage of the outflow leaves by diffusion through the iris, ciliary body, and choroid via venous drainage (unconventional outflow) (5). Decreased aqueous humor outflow occurs due to physiologic or structural modifications of the outflow pathways from the posterior chamber, pupil, filtration angle, trabecular meshwork (TM), vasculature of the scleral venous plexus, and choroidal outflow pathways (6–8).

Canine glaucoma may be classified into three basic categories: congenital, primary, and secondary. Congenital or early-onset glaucoma manifests *in utero*, in neonatal animals, or early in life (9–14). This form of glaucoma is usually associated with ASD, which manifests with ocular anomalies, including hypoplastic filtration angle and ciliary cleft, lenticular and anterior uveal hypoplasia, and cataracts (9–11).

Primary glaucoma is most commonly associated with goniodysgenesis and occurs in several breeds of dogs (15). Goniodysgenesis is a synonym for pectinate ligament dysplasia (PLD), where there is limited fenestration of the neural crest tissues within the iridocorneal angle during ocular development (15). This form of glaucoma manifests in mid- to late-life and initially presents in one eye with the contralateral eye developing glaucoma thereafter (months to years) (16). A genetic predisposition is assumed for primary glaucoma associated with goniodysgenesis (15, 17). Goniodysgenesis is considered to be a marker for primary glaucoma, as only a small percentage (<10%) of dogs with this anomaly will develop glaucoma in mid- to late-life (15).

Secondary glaucoma is an acquired condition and develops when there is antecedent ocular pathology, resulting in reduced aqueous humor drainage. Secondary glaucoma may be unilateral or bilateral depending on the etiology (8).

A common histologic finding in all types of canine glaucoma is the presence of pre-iridal membranes that may span the filtration angle. In 1990, Peiffer et al. initially described the presence of these membranes in 83 diseased canine eyes. The membranes were described as monocellular, vascular, or fibrous. It was proposed that these membranes formed from the iridal tissues in response to angiogenic and fibroblastic cytokines released from intraocular tissues in response to chronic inflammation, neoplasia, or retinal ischemia (18). Pre-iridal membranes were most commonly noticed in globes with chronic retinal detachments, endophthalmitis, chronic glaucoma, and neoplasia (ciliary body adenoma, uveal melanoma, and metastatic tumors) (19). In a study examining the light microscopic appearance of the iridocorneal angle (ICA) in chronic glaucoma, it was noted that pre-iridal monocellular membranes were more common in eyes diagnosed with primary glaucoma associated with goniodysgenesis, whereas fibrovascular membranes were more common in secondary glaucoma (18).

It has been suggested that pre-iridal monocellular membranes may represent an early form of the pre-iridal fibrovascular membrane (PIFM) or that they may originate from the metaplastic corneal endothelium that traverses the filtration angle (3, 19, 20).

Recently, it was published that pre-iridal monocellular membranes are a normal histologic finding in dogs and that they become metaplastic in the presence of glaucoma, and although they are present in each of the pre-mentioned categories of glaucoma, they are easiest to detect in primary glaucoma (21). Based on these findings, it is appropriate to review these membranes in a larger number of canine globes with glaucoma.

To the best of our knowledge, there are no publications that have studied the prevalence of monocellular or fibrovascular membranes in canine globes, including all categories of glaucoma.

The objectives of this study were to (i) evaluate the prevalence of monocellular and fibrovascular pre-iridal membranes in dogs diagnosed with congenital/ASD-associated glaucoma, dogs with primary glaucoma associated with goniodysgenesis, and those with secondary glaucoma and (ii) determine whether there is any association between the presence of monocellular and fibrovascular pre-iridal membranes from light microscopic examination findings and breed, gender, age, histopathological ocular changes of glaucoma.

## Materials and methods

### Data collection

All enucleated canine globes that had been submitted to the Ocular Pathology Service over an 8-year period (2000–2018) due to glaucoma were reviewed.

Patient information collected included the following: clinical diagnosis and ophthalmic examination findings, breed, gender, neuter status, and age at presentation. Only cases examined clinically by a board-certified veterinary ophthalmologist were included in the study. Periodic acid–Schiff and/or hematoxylin and eosin (H&E)-stained slides were reviewed with light microscopy. The slides of each enucleated globe were re-examined by an ophthalmology resident, a board-certified veterinary ophthalmologist (DACVO), and a pathologist (DACVP) for the purposes of this study.

The minimum inclusion criteria for dogs to be considered to have congenital/ASD-associated glaucoma included an age of ≤3 years with buphthalmos and ophthalmic and histologic findings of glaucoma and ASD (such as ciliary body, iris, and filtration angle hypoplasia, microphakia, cataract, and elongated hypoplastic ciliary processes).

The inclusion criteria for primary glaucoma included gonioscopic examination of all the globes confirming goniodysgenesis in the non-glaucomatous eye and, when at least 1-year follow-up was possible, confirmation of glaucoma in the contralateral eye that resulted in enucleation (*n* = 5 dogs) and light microscopic examination of both globes that excluded antecedent ocular disease (*n* = 8 globes).

The inclusion criteria for secondary glaucoma included globes with the common predisposing intraocular disease (intraocular neoplasia, pigmentary uveitis, melanocytosis of Cairn Terriers, lens luxation, endophthalmitis, glaucoma secondary to phacoemulsification, and lens-induced uveitis) confirmed on clinical and light microscopic examinations.

### Light microscopic evaluation

Archived enucleated globes, which had been formalin- or Davidson’s fixed and paraffin-embedded, were routinely sectioned with a minimum of one 4-μm sagittal section through the pupil, and the optic nerve was stained with hematoxylin–eosin (H&E) and periodic acid–Schiff (PAS). Histologic features were recorded for each globe including: the type of neoplasia in secondary glaucoma, corneal striae, corneal ulcer, corneal edema, closed ciliary cleft (CC), peripheral anterior synechia (PAS), type of uveal inflammation, uveal cysts (classified as thin-walled cysts of the Golden Retriever, related to posterior iris epithelium atrophy or ciliary process cyst), uveal atrophy, anterior or posterior synechia, cataract, tapetal sparing, retinal detachment (classified as exudative or serous when there was an accumulation of fluid within the subretinal space such as serous effusion, fresh blood, or inflammatory exudates), rhegmatogenous retinal detachment (when a full-thickness hole or tear was evident in the neurosensory retina with vitreous in the subretinal space), focal retinal detachment when the focal areas of retinal pigment epithelium (RPE) hypertrophy with the loss of the outer nuclear layer were noted, and retinal degeneration that reduced the tissue to a glial scar.

The ICA was described as being open if there was no apposition of the iris base to the peripheral cornea and close if there was peripheral anterior synechiae (PAS), in which the iris was attached to the corneal endothelium. Angle recession was noted if the ICA was stretched so that the iris base was distanced from the termination of Descemet’s membrane and pectinate ligaments were flattened against the inner sclera. The ciliary cleft was described as open or collapsed.

**Figure 1:**
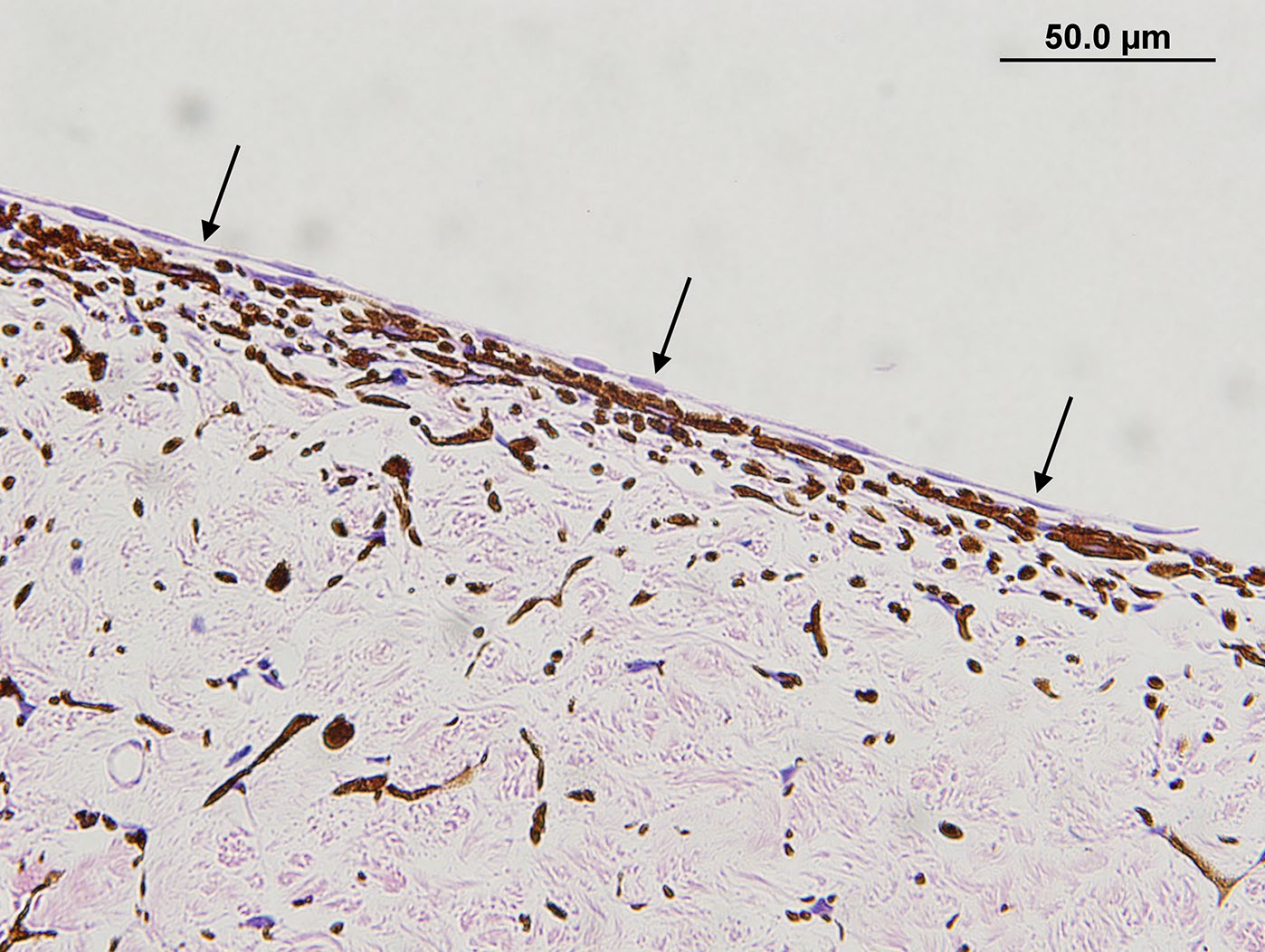
Monocellular membrane in a dog with primary glaucoma. Note the monolayer of plump to spindle cells that are extending over the entire anterior iris surface (black arrows) (hematoxylin & eosin stain).

**Figure 2:**
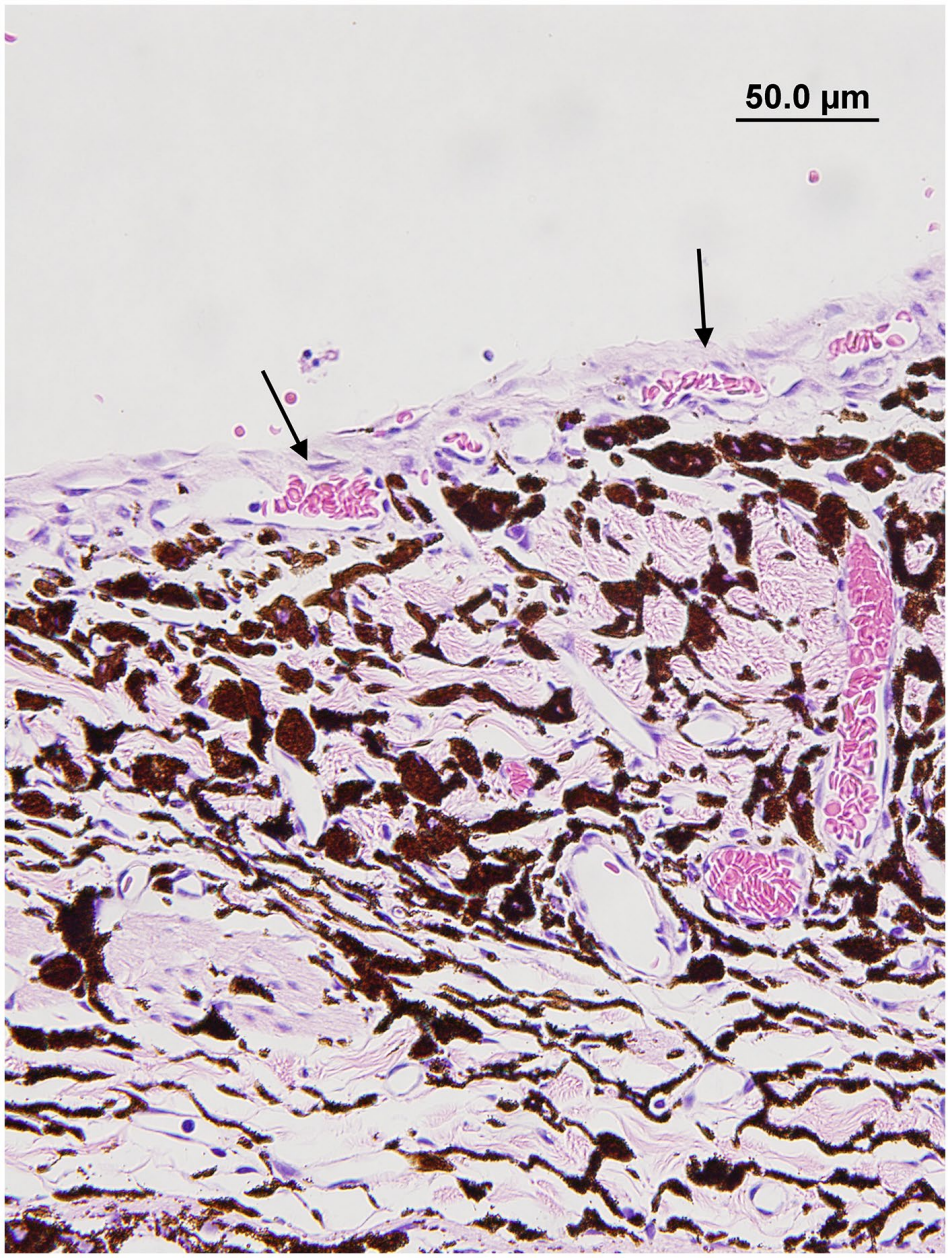
Pre-iridal fibrovascular membrane membrane in a dog with secondary glaucoma. Note how the iridal blood vessels are expanding and covering the anterior iris surface (black arrows) (hematoxylin & eosin stain).

The presence and type of pre-iridal membrane (monocellular or fibrovascular) were also recorded. The criterion for a monocellular membrane was a single layer of multiple contiguous non-pigmented plump and flattened spindle cells that were closely apposed or slightly lifted from the anterior surface of the iris (Figure 1). The criterion for a PIFM was a vascular/fibrous membrane that covered the surface of the iris, formed from small blood vessels that arose from the underlying iridal vessels and fibrous tissue (Figure 2).

### Statistical analysis

Fisher’s exact test was used to compare the prevalence of the presence of membranes across congenital glaucoma/ASD, primary glaucoma associated with goniodysgenesis, and secondary glaucoma, as well as the presence of each membrane type. Odds ratios were calculated as a measure of clinical effect for each of the histological lesions compared to globes with monocellular vs. fibrovascular membranes. The non-parametric *t*-test was used to evaluate the prevalence of membranes within age and gender and to compare the age of dogs with congenital/ASD-associated glaucoma with primary or secondary glaucoma and between dogs with primary and secondary glaucoma.

Statistical significance for all tests was set at a *p*-value ≤ 0.05, and Stata14 software (StataCorp) was used for data analyses. If dogs had more than one globe included within the study, one eye was excluded from the statistical dataset to ensure that all observations were independent within the statistical analysis. Only globes with the same type of membrane on each side of the iris leaflet were included in the analyses to avoid bias (*n* = 105).

## Results

### Association of monocellular and fibrovascular pre-iridal membranes with type of glaucoma

A total of 108 canine globes (101 dogs) were included in the study. Of those 108 globes, 19 (19 dogs) were diagnosed with congenital/ASD-associated glaucoma, 37 (40 dogs) with primary glaucoma associated with goniodysgenesis, and 46 (49 dogs) with secondary glaucoma (7 intraocular neoplasia, 5 pigmentary uveitis, 2 melanocytosis of Cairn Terriers, 3 primary lens luxation, 10 idiopathic uveitis, 4 glaucoma secondary to phacoemulsification, 6 lens-induced uveitis, 4 infected corneal ulcers, and 8 retinal detachments). One eye with primary glaucoma had a fibrovascular membrane on one side of the iris leaflet and a monocellular on the other side, and this finding was excluded from the statistical analysis. The prevalence of pre-iridal membranes in each glaucoma category is summarized in Table 1.

**Table 1:** Prevalence of membranes in each type of glaucoma.

Glaucoma	Congenital/ASD	Primary	Secondary	*p*-value
Total	19	40 (39)*	49 (47)*	108 (105)*
Presence of membrane	13/19 (68%)	36/39 (92%)	43/47 (91%)	0.033
Monocellular	10/19 (53%)	29/39 (74%)	22/47 (47%)	0.037
Fibrovascular	3/19 (16%)	8/39 (20%)	22/47 (47%)	0.010

^*^For the *p*-value only, globes with the same type of membrane on each side were included to avoid bias (*n* = 105).

There was a significant difference in the proportion of globes with any type of membrane across the three glaucoma categories (*p* = 0.033). There was also a significant difference in the proportion of globes with monocellular membranes across the three glaucoma categories (*p* = 0.037) likely driven by the higher proportion (74%) in the primary glaucoma category. There was a significant difference in the proportion of globes containing fibrovascular membranes across the three glaucoma categories (*p* = 0.01) likely driven by the moderately higher proportion of fibrovascular membranes in the secondary glaucoma category (47%).

There was a significant difference in the proportion of globes with monocellular membranes across the three glaucoma categories (*p* = 0.04) likely driven by the higher proportion of globes with monocellular membranes detectable in the primary glaucoma category (74%) compared to ASD/congenital (53%) and secondary glaucoma (47%). There was also a significant difference in the proportion of globes containing fibrovascular membranes across the three glaucoma categories (*p* = 0.01) likely driven by the relatively higher proportion of fibrovascular membranes in the secondary glaucoma category (47%) than ASD/congenital (16%) and primary glaucoma (20%).

Monocellular membranes were significantly more common than fibrovascular membranes in the primary glaucoma group (*p* < 0.001). There were no significant differences in the presence of monocellular versus fibrovascular membranes in the congenital/ASD group (*p* = 0.09) or the secondary glaucoma group (*p* = 1.0).

### Associations of demographics with type of glaucoma and pre-iridal membranes

The most representative breeds in the congenital/ASD-associated glaucoma group (*n* = 19) were the Siberian Husky (*n* = 3) and German Shepherd (*n* = 2); however, these numbers were too small to be meaningful.

In primary glaucoma associated with goniodysgenesis (*n* = 37), the most common breeds were Cocker Spaniel (*n* = 6), Shih Tzu (*n* = 5), Labrador Retriever (*n* = 5), and Shiba Inu (*n* = 5).

The most common breeds included in secondary glaucoma (*n* = 46) were Cocker Spaniel (*n* = 8), Golden Retriever (*n* = 5), Shih Tzu (*n* = 4), and Boston Terrier (*n* = 4). We could not assess the statistical associations between breed and type of glaucoma or the presence of a pre-iridal membrane due to low statistical power.

In the congenital/ASD-associated glaucoma group, 9 were female and 5 were male dogs, and in 4 dogs, the gender was not recorded in the medical records. In the primary glaucoma associated with the goniodysgenesis group, there were 17 female and 20 male dogs. In the secondary glaucoma group, there were 25 female and 21 male dogs. There was no statistical difference with sex and the presence of a pre-iridal monocellular (*p* = 0.98) or fibrovascular membrane (*p* = 0.5).

The median age of dogs with congenital/ASD-associated glaucoma was 1.3 years (range 0.3–3), those with the primary glaucoma associated with GD was 8.3, and those with secondary glaucoma was 8.9. As expected, the median age of dogs with congenital/ASD-associated glaucoma was significantly younger when compared to dogs with primary or secondary glaucoma (*p* < 0.0001). Age was not significantly different between dogs with primary and secondary glaucoma (*p* = 0.38). There was no association between age and presence of a pre-iridal membrane in either the monocellular (*p* = 0.76) or fibrovascular membrane groups (*p* = 0.90).

### Association of pre-iridal membranes with light microscopic findings

Monocellular membranes are a continuous sheet of spindle cells with scattered round cells that extend over the anterior iris face, whereas fibrovascular membranes consist of multiple layers of small vessels arising from the anterior stroma of the iris.

Associations between monocellular and fibrovascular membranes with light microscopic findings and type of glaucoma are summarized in Tables 2, 3. The most common light microscopic findings overall were a collapsed ciliary cleft, which was present in 72% of globes, and retinal degeneration involving both the inner and outer retina (glial scar formation), which was present in 69% of globes.

**Table 2:** Association between histologic findings and type of membrane.

Histologic findings	Monocellular membrane (62 eyes)	Fibrovascular membrane (36 eyes)	*p*-value	Odds ratio (95% confidence interval)
Corneal striae	2 (3%)	2 (5%)	0.59	0.58 (0.04–8.42)
Corneal ulcer	5 (8%)	3 (8%)	1	1 (0.18–6.8)
Corneal edema	31 (50%)	15 (40%)	0.36	1.4 (0.6–3.6)
CC: close	42 (68%)	28 (76%)	0.40	0.67 (0.23–1.8)
PAS	3 (5%)	13 (35%)	0.0001	0.094 (0.02–0.39)
Uveal cyst	9 (15%)	5 (13%)	0.89	1 (0.3–4.5)
Uveal inflammation	25 (41%)	13 (38%)	0.61	1.2 (0.5–3.2)
Uveal atrophy	24 (38%)	6 (16%)	0.02	3.26 (1.1–10.9)
AS	12 (19%)	9 (24%)	0.56	0.75 (0.25–2.3)
PS	9 (15%)	6 (16%)	0.82	0.87 (0.25–3.3)
Cataract	21 (34%)	19 (51%)	0.08	0.48 (0.2–1.2)
Retinal detachment	14 (22%)	12 (32%)	0.28	0.6 (0.22–1.7)
Retinal degeneration (glial scar)	39 (63%)	26 (70%)	0.45	0.7 (0.26–1.85)
Dorsal/tapetal sparing	19 (31%)	6 (16%)	0.11	2.3 (0.75–7.7)
Vitreous degeneration	2 (3%)	2 (5%)	0.60	0.58 (0.40–8.4)
Angle recession	9 (14%)	3 (8%)	0.34	1.9 (0.43–11.75)

CC, ciliary cleft; AS, anterior synechia located elsewhere than the base of the iris (PAS); PS, posterior synechia.

**Table 3:** Histological findings associated with each type of glaucoma.

	Congenital			Primary			Secondary			Total
# eyes cases	19			40			49			108
Corneal striae	3 (17%)			1 (3%)			1 (2%)			5 (4.6%)
Corneal ulcer	1 (5.5%)			3 (7.5%)			4 (9%)			8 (7.4%)
Corneal edema	5 (28%)			21 (58%)			24 (49%)			50 (46%)
CC: collapsed	11 (58%)			35 (87.5%)			32 (65%)			78 (72%)
PAS	0 (0%)			3 (7.5%)			13 (26.5%)			16 (15%)
Uveal cyst	1 (5.5%)			5 (13.8%)			9 (19.5%)			15 (14%)
	TW	CP	A	TW	CP	A	TW	CP	A	
	0	0	1 (100%)	0	2 (40%)	3 (60%)	5 (55%)	1 (11%)	3 (33%)	
	4 (22.2%)			15 (41.6%)			22 (47.8%)			
Uveal inflammation	L	LP	LPN	L	LP	LPN	L	LP	LPN	
	0	4 (100%)	0	0	14 (93%)	1 (7%)	2 (9%)	13 (59%)	7 (32%)	41 (38%)
Uveal atrophy	18 (100%)			13 (36%)			6 (13%)			
AS	5 (27%)			6 (17%)			15 (33%)			26 (24%)
PS	2 (11%)			8 (20%)			7 (15%)			17 (16%)
Cataract	5 (27%)			9 (25%)			27 (55%)			41 (38%)
Retinal detachment	5 (33%)			6 (11%)			17 (39%)			28 (26%)
	S/E	F	R	S/E	F	R	S/E	F	R	
	1 (20%)	0	4 (80%)	2 (33%)	4 (66%)	0	7 (41%)	8 (47%)	2 (12%)	
Retinal degeneration (glial scar)	15 (79%)			26 (65%)			33 (67%)			74 (69%)
Dorsal/tapetal sparing	1 (5.5%)			18 (45%)			7 (15%)			26 (24%)
Angle recession	1 (5.5%)			6 (17%)			8 (17%)			15 (14%)

CC, ciliary cleft; PAS, peripheral anterior synechia; AS, anterior synechia located elsewhere than the base of the iris (PAS); PS, posterior synechia; L, lymphocytic uveitis; LP, lymphoplasmacytic uveitis; LPN, lymphoplasmacytic neutrophilic uveitis; TW, thin-walled cyst; CP, ciliary process cyst; A, posterior iris epithelium atrophy; S, serous; E, exudative; F, R, focal (area of RPE hypertrophy without serous component), rhegmatogenous; NA, not applicable.

There was a significant statistical difference in the proportion of PAS (*p* = 0.0001) across monocellular and fibrovascular membranes. The odds of PAS were 0.094 (CI = 0.02–0.39) times less likely for globes with monocellular membranes than in globes with fibrovascular membranes. There was also a statistically significant difference in the proportion of globes with uveal atrophy (*p* = 0.02) across monocellular and fibrovascular membranes. The odds of uveal atrophy were 3.26 (CI = 1.1–10.9) times more likely in globes with monocellular membranes than in fibrovascular membranes. There were no other significant associations between light microscopic findings and type of pre-iridal membrane.

## Discussion

Light microscopic examination of glaucomatous eyes revealed pre-iridal membranes present in the majority 98/108 (91%) of the diseased canine globes. Of these, most 62/98 (63%) were monocellular, while 36/98 (37%) were fibrovascular membranes.

Peiffer et al. were the first to describe the presence of a monocellular membrane in the anterior surface of the iris. Monocellular membranes were described as a monolayer of plump cells and occasionally spindle cells that extended over the anterior iris surface (19). However, these were not quantified or evaluated with respect to disease type. Bauer et al. described monocellular membranes in 12 glaucomatous globes, with 8 present in globes with primary glaucoma and 5 with secondary glaucoma (18). We noted monocellular membranes to be present in 61 glaucomatous globes and confirmed their association with primary glaucoma, as 74% of globes with primary glaucoma had a pre-iridal monocellular membrane.

Although monocellular membranes were more common in the primary glaucoma group compared to other categories of glaucoma, they were not exclusive to this group, as they were a common histologic finding in all forms of glaucoma. This included 47% of globes with secondary glaucoma and 54% of globes with ASD-associated glaucoma.

Monocellular membranes were recently reported in normal dogs, and immunohistochemical changes were documented in these when glaucoma was present (21). It is important to understand that although all normal canine globes have a pre-iridal monocellular membrane, they are not always detectable with light microscopy, which explains why they are not always noted histologically within glaucomatous globes (21) Furthermore, when marked inflammation, anterior uveal neoplasia and PIFMs are present it is very challenging to detect a monocellular membrane on the iris surface, which explains why monocellular membranes are more commonly detected in primary goniodysgenesis associated glaucoma compared to other forms of glaucoma (21).

The most common light microscopic findings in globes with all types of glaucoma were a collapsed ciliary cleft and retinal degeneration. These findings are supported by the veterinary literature (3, 7, 22). Elevated IOP leads to retinal degeneration secondary to ganglion cell necrosis, apoptosis, and axonal compression within the optic nerve (3, 23). The ciliary cleft is collapsed in most dogs presenting with chronic glaucoma; however, the exact mechanism by which this occurs is unknown (11). Primary glaucoma in our opinion is simply the compression of the delicate filtration tissues by the increase in the IOP. In secondary glaucoma, the presence of neoplastic, inflammatory cells, and fibrovascular membranes occlude or collapse the ciliary cleft (24).

When light microscopic findings were evaluated for associations with membrane type, only PAS and uveal atrophy showed any association. PAS is described as an adhesion of the anterior surface of the iris root with the peripheral cornea causing closure of the iridocorneal angle (10). PAS was 10.7 times (CI = 2.5–61.8) more likely to be present in globes with fibrovascular membranes. PAS is reported to be a common finding in globes with fibrovascular membranes and likely occurs secondary to the fibrovascular membrane extending across the corneal endothelium effectively closing the iridocorneal angle (25). Uveal atrophy is described as a decrease in thickness secondary to loss of pigment, smooth muscle, and vascular tissue (10). Uveal atrophy is common with all categories of chronic glaucoma, and it is the direct result of tissue hypoxia and elevated pressure causing a loss of pigment, smooth muscle, and vasculature (15). Uveal atrophy was more likely to be detected in globes with monocellular membranes and less neoplastic and inflammation that masked the presence of uveal atrophy in secondary glaucomas.

The significance of age in dogs with congenital/ASD glaucoma (*p* < 0.0001) is not surprising as clinical signs and structural abnormalities typical of this type of glaucoma are present at birth or early in life. Both primary and secondary glaucoma had a median age of around 8 years, which is similar to previous reports (26, 27).

The limitations of this study are those common to all retrospective studies, including incomplete clinical and histopathological information, which reduced the number of animals that met our inclusion criteria. We were unable to truly assess the severity or duration of glaucoma prior to enucleation due to difficulties confirming historical information. Moreover, with chronic glaucoma, some changes that can be seen histologically include an alteration in the filtration angle morphology (7). Some dogs with primary glaucoma were lost to follow-up, and thus, we were unable to confirm whether they developed glaucoma in the contralateral eye.

Most eyes included in this study were enucleated at a very late stage in the disease process, and many of the observations seen may be secondary to extensive periods of elevated intraocular pressure and medical and/or surgical therapy.

Primary lens luxation secondary to zonular degeneration will lead to the development of glaucoma resulting from the vitreous being displaced into the anterior chamber with the obstruction of the pupil and iridocorneal angle (6). We classified primary lens luxation under the secondary glaucoma category. As genetic testing for the mutation in the ADAMTS10 and ADAMTS17 genes was not performed, the possibility of primary open-angle glaucoma in some of these cases cannot be excluded.

Finally, gonioscopy was performed neither in all secondary glaucoma cases nor when glaucoma presented simultaneously bilateral cases.

In summary, pre-iridal monocellular membranes are common and are present in all forms of glaucoma; however, they can be difficult to identify histologically. Monocellular membranes are easiest to detect histologically when there is minimal inflammation, PIFMs, or neoplastic anterior uveal disease and therefore most commonly detectable in primary goniodysgenesis-associated glaucoma compared to secondary glaucoma where fibrovascular membranes predominate and conceal the monocellular membranes.

## Data availability statement

The raw data supporting the conclusions of this article will be made available by the authors, without undue reservation.

## Ethics statement

Ethical approval was not required for the studies involving animals in accordance with the local legislation and institutional requirements because the medical records of dogs referred to the ophthalmology service and were an enucleated globe(s) submitted to an Ocular Pathology Service. Samples were reviewed from the stored archive. Written informed consent was obtained from the owners for the participation of their animals in this study.

## Author contributions

LB: Writing – original draft. LS: Writing – review & editing, Supervision. JC: Formal analysis, Writing – review & editing. BG: Supervision, Visualization, Writing – review & editing.
